# Editorial: Translational control in cancer

**DOI:** 10.1093/narcan/zcae031

**Published:** 2024-07-23

**Authors:** Francesca Aguilo, Erik Dassi

**Affiliations:** Department of Molecular Biology, Umeå University, SE-901 85 Umeå, Sweden; Wallenberg Centre for Molecular Medicine, Umeå University, SE-901 85 Umeå, Sweden; Laboratory of RNA Regulatory Networks, Department of Cellular, Computational and Integrative Biology (CIBIO), University of Trento, Via Sommarive 9, 38123 Trento (TN), Italy

Translational control is crucial to support the increased anabolic demands of cancer cells and thus for cancer development and progression. Boosting oncogenic translation is a multifaceted process. It involves changes in ribosomal biogenesis, including changes in ribosomal composition through differential incorporation of ribosomal proteins and post-transcriptional modifications. Translational control in cancer also involves changes in the dynamic interplay between RNA-binding proteins (RBPs), RNA structural elements, and non-coding RNAs. These mechanisms affect both global protein synthesis and selective translation of specific mRNAs to promote tumor cell survival, angiogenesis, transformation, invasion and metastasis. Alterations in translational control vary with the type of cancers, the disease stage, and the tumor microenvironment. These factors underscore both the importance of understanding translation regulation in cancer and the possibility of identifying novel therapeutic targets. *NAR Cancer* is pleased to publish a collection of 11 review articles approaching the topic of translational control in cancer from different perspectives.

Translation involves an interplay among ribosomes, transfer RNAs (tRNAs), amino acids, eukaryotic initiation factors (eIFs) and eukaryotic elongation factors (eEFs). These work in harmony to promote translation of the mRNA into protein. Among the sequential stages of translation—initiation, elongation, termination and ribosome recycling—initiation is the rate-limiting step, with the assembly of the translation initiation complex (eIF4F) emerging as the critical process. Cap-dependent translation, a stringently regulated process that is essential for synthesis of most cellular proteins, involves the interaction of the eIF4F complex, composed of eIF4E, eIF4G and eIF4A, with the 7-methylguanosine mRNA cap. This initiates assembly of the translation machinery at the 5′ end of the mRNA, facilitating ribosome recruitment and subsequent protein synthesis. The binding of eIF4G and poly(A)-binding protein (PABP) at the 3′ polyadenylated end of the mRNA further enhances the stability of the translational complex.

In recent years, research has revealed specialized, eIF4E-independent translation mechanisms that are used under stress conditions. Specifically, during tumor development, cells can globally reduce conventional eIF4E/cap-mediated translation and instead use alternative, non-canonical translational initiation mechanisms. These include: (i) internal ribosome entry site (IRES)-mediated mRNA translation; (ii) cap-dependent, but eIF4E-independent mechanisms using eIF4E paralogs or an alternate cap-binding protein such as eIF3d and (iii) cap-independent translation enhancer-mediated binding of translation initiation factors to the 5′-untranslated region (5′UTR). Mahé *et al*. provide a detailed review of these **non-canonical mRNA translation** initiation mechanisms in cell stress and cancer (1).

**Ribosomal heterogeneity**, the existence of structurally and functionally diverse ribosomes within a cell, is emerging as a critical factor in the regulation of oncogenic translation. This diversity arises from variations in ribosomal RNA (rRNA) modifications, differential expression of ribosomal proteins, incorporation of alternative ribosomal components, and post-translational modifications of ribosomal proteins. Such heterogeneity allows cells to fine-tune protein synthesis, selectively translating specific subsets of mRNAs in response to developmental cues, environmental changes, and stress conditions. In the context of cancer, alterations in ribosomal composition and function are often observed, contributing to the uncontrolled growth and proliferation of tumor cells. Ramalho *et al*. bring insight into the role of **specialized ribosomes** in cancer and how these mechanisms may be exploited for cancer-selective therapeutics (2).

Also delving into the striking complexity of the ribosomal machinery, Fuentes *et al*. highlight how ribosomal proteins contribute to ribosome biogenesis and how alterations to this process can lead to the formation of **onco-ribosomes** (3). They describe how mutations in these proteins can drive clonal evolution and give rise to therapeutic resistance in cancer. **Translational reprogramming** is also made possible for cancer cells by hijacking ribosome biogenesis. Hwang and Denicourt introduce us to how this process is indeed tightly intertwined with cancer hallmarks (4). They highlight that, from stemness maintenance and the epithelial-mesenchymal transition to stress response, oncogenic signaling pathways are heavily affected by the alteration in this critical cellular process, also presenting relevant therapeutic opportunities.

**Chemical modifications of RNA** play a crucial role in all stages of physiologic and oncogenic translation. During translation initiation, these modifications facilitate the recruitment of ribosomes to the start codon, thereby enhancing translation efficiency. They also shape the RNA’s secondary structure, impacting the accessibility of ribosomes and initiation factors to the start codon. In the elongation phase, modifications on transfer RNAs (tRNAs) and rRNAs are critical for accurate codon recognition. At the termination step, they are essential for recognizing the stop codon and ensuring the proper release of the newly synthesized protein.

**N6-methyladenosine (m^6^A)**, the most abundant internal modification on mRNA, can impact several aspects of translation depending on its location. For instance, in the 3′UTR, an m^6^A reader protein, YTHDF1, recognizes m^6^A modifications and, through mRNA looping, facilitates interaction with eIF3 to initiate translation. The deposition of the m^6^A mark on certain mRNAs has been largely associated with uncontrolled proliferation and survival of cancer cells. Among these, MYC is a prominent m^6^A-modified oncogene, characterized by a significant translation upregulation during tumorigenesis. Other oncogenic pathways such as Wnt/β-catenin, PI3K/AKT/mTOR and RAS/RAF/MEK/ERK have also been linked to m^6^A-mediated translation. Focusing on m^6^A, Esteva-Socias and Aguilo detail its role in translation, including the non-canonical function of METTL3 in promoting translation independently of m^6^A deposition (5).

**tRNA modifications** impact protein synthesis by promoting the production of proteins that drive tumor growth, characterized by specific codon usage patterns in their mRNAs. This process, termed codon-biased translation, favors the translation of certain codons due to increased tRNA availability or stability. It was first observed in stress response regulation and is proposed as modification-tunable transcripts (MoTTs), enhancing translation efficiency in stress-related protein transcripts. Alterations in tRNA modification levels at wobble bases can affect the degeneracy of the genetic code, leading to translation defects like mistranslation, codon-specific translation pauses, or changes in translation speed. Additionally, chemical modifications in tRNA backbone regions, particularly in the D- and T-loops, support tRNA folding and stability, indirectly influencing tRNA processing and protein synthesis regulation. Although these modifications do not directly impact mRNA recognition by tRNA, they play a crucial role in modulating protein synthesis through indirect mechanisms. Añazco-Guenkova *et al*. describe how tRNA modifications can contribute to tumorigenesis (6).

**Small nucleolar RNAs (snoRNAs)**play a crucial role in the modification and processing of rRNA, which is essential for proper ribosome function and, consequently, translation. snoRNAs guide chemical modifications of rRNA, including 2′-*O*-methylation and pseudouridylation, which are critical for the structural stability and functional competence of ribosomes. To support cell growth and proliferation, cancer cells modulate the expression of all the components involved in ribosomal biogenesis to meet the increased demand for ribosomes and sustain hyperactive protein synthesis. Numerous studies have shown the correlation between alterations in the expression of specific snoRNAs and various cancer features, such as proliferation, migration, and invasiveness of cancer cells. This suggests that snoRNAs could serve as potential biomarkers for the assessment of cancer diagnosis and prognosis. In this issue, Zacchini *et al*. provide a comprehensive review of snoRNA function in cancer (7).

Translation can also be modulated by the presence of specific sequence or **structural regulatory elements**. Among these elements are binding sites for **RNA-binding proteins (RBPs)**, a thousand-strong class of proteins which preside over post-transcriptional regulation of gene expression. RBPs have recently been the focus of considerable attention in cancer, due to their involvement in many tumor processes through their modulation of translation. Ciocia *et al*. discuss here the functions and mechanism of action of CSDE1, one such RBP with a key role in controlling cancer properties (8).

Structural and sequence elements in mRNA also regulate translation. These include terminal oligopyrimidine tracts, internal ribosome entry sites, **upstream open reading frames (uORFs)** and RNA G-quadruplexes (RG4s). The uORFs are regions upstream of the main protein coding sequence where ribosomes can assemble to produce distinct peptides or affect translation initiation of the canonical ORF. Dasgupta and Prensner describe how uORFs and their 5′UTR sequences can modulate oncogenic signaling and tumor-immune interactions through these types of mechanisms (9). **RNA G-quadruplexes**are widely-distributed, non-canonical, four-stranded structures that control multiple steps of post-transcriptional and translational gene expression regulation. They are involved in the pathology of cancer and other diseases. Cammas *et al*. focus on how RG4s specifically contribute to the translational reprogramming occurring in cancer cells through paradigmatic examples highlighting their role (10).

Finally, the possibility to understand the role of translation in cancer has and continues to be enabled by **technological advances** expanding our capabilities to measure key molecular components of the cell. Next-generation sequencing has allowed a considerable leap forward in how we assess mRNA translation and its regulatory dynamics. In this issue, Román *et al*. lead us to understand how this cornerstone technology has led to the development of many fundamental techniques able to address various aspects of aberrant mRNA translation in cancer cells (11).
